# Disability 4.0: bioethical considerations on the use of embodied artificial intelligence

**DOI:** 10.3389/fmed.2024.1437280

**Published:** 2024-08-16

**Authors:** Francesco De Micco, Vittoradolfo Tambone, Paola Frati, Mariano Cingolani, Roberto Scendoni

**Affiliations:** ^1^Research Unit of Bioethics and Humanities, Department of Medicine and Surgery, University Campus Bio-Medico of Rome, Rome, Italy; ^2^Operative Research Unit of Clinical Affairs, Healthcare Bioethics Center, Fondazione Policlinico Universitario Campus Bio-Medico, Rome, Italy; ^3^Department of Anatomical, Histological, Forensic and Orthopedic Sciences, Sapienza University, Rome, Italy; ^4^Department of Law, Institute of Legal Medicine, University of Macerata, Macerata, Italy

**Keywords:** impairment, disability, handicap, robotics, artificial intelligence, medical ethics

## Abstract

Robotics and artificial intelligence have marked the beginning of a new era in the care and integration of people with disabilities, helping to promote their independence, autonomy and social participation. In this area, bioethical reflection assumes a key role at anthropological, ethical, legal and socio-political levels. However, there is currently a substantial diversity of opinions and ethical arguments, as well as a lack of consensus on the use of assistive robots, while the focus remains predominantly on the usability of products. The article presents a bioethical analysis that highlights the risk arising from using embodied artificial intelligence according to a functionalist model. Failure to recognize disability as the result of a complex interplay between health, personal and situational factors could result in potential damage to the intrinsic dignity of the person and human relations with healthcare workers. Furthermore, the danger of discrimination in accessing these new technologies is highlighted, emphasizing the need for an ethical approach that considers the social and moral implications of implementing embodied AI in the field of rehabilitation.

## Introduction

Disability is the result of a complex relationship between an individual's health condition, personal factors and environmental factors representing the circumstances in which the human being lives (1). The definition of disability reflects the impact that an impairment of function or body may have on a person's activities, also in relation to the environment that may be a barrier or facilitator (2). This involved moving away from the functionalist model, based on a conception of the human body as a material entity separate from the personal dimension to a holistic approach that conceives disability as the result of the complex interaction between biological, psychological, social, and environmental factors. This interaction, in addition to shaping the individual experience of the person with disabilities and the level of challenge it implies, has catalyzed significant changes in intervention protocols. This approach promoted the empowerment and autonomy of people with disabilities, encouraging their active participation in social life (3, 4).

The introduction of robotics and artificial intelligence (AI) has opened a new chapter in the care and integration of people with disabilities, facilitating their independence, autonomy, and social participation (5). After central nervous system injuries that impair motor coordination, recovery of motor functions and skills requires repetition of movements in the affected part and stimulation of brain plasticity. Robotics for rehabilitation purposes facilitates guided movement of the upper and lower limbs, optimizing therapeutic and functional effects. These technologies provide feedback to patients, allowing them to adjust the strength and maximize the effectiveness of therapy, accelerating the recovery process (6–10). Robot-assisted rehabilitation offers muscle support therapies and repetition of basic motor activities, enabling users to perform them comfortably in the home environment through integration with personal computers, often using technologies originally developed for other purposes, such as games (11–13). Therapeutic robots, capable of simulating social interactions such as communication and play, help patients with dementia, Alzheimer's, autism, and childhood motor disabilities (14–17).

Robots in medicine speed up operations, improve diagnosis, and increase efficiency. They also bring care to those who are far away or have financial difficulties. But they pose new ethical and legal challenges that must be addressed carefully (18). The recent speedy progress of robotics deserves an articulated ethical reflection with respect to the real present problems and the anticipation of probable or possible future scenarios (19). In this specific area, bioethical reflection assumes a key role on various levels: on an anthropological dimension, defining the concept of corporeity and the challenges associated with physical impairments; on an ethical dimension, establishing guiding principles for rehabilitation programmes and those involved in them, as well as considering the autonomy of people with disabilities; on a legal dimension, recognizing the rights of people with disabilities as autonomous subjects; and on a socio-political dimension, guiding the distribution of resources in the health care sector and promoting community inclusion through appropriate project interventions (20). Nevertheless, as things stand, there is a lack of an ethical framework to underpin the interaction between disabled people and AI, while the focus is mainly on the usability of products (21).

The purpose of this article is to examine in detail the ethical issues that arise from the increasingly widespread use of robotics and AI in the context of disabilities. The authors want to discuss the moral and social implications of these technologies for disabled people, exploring issues such as autonomy, dignity, equity in access to services and responsibility in the implementation and use of these technologies. They also aim to provide ethical recommendations and practical suggestions to ensure the responsible and respectful use of robotics and AI in improving the lives of people with disabilities.

## The principle of autonomy and respect for the intrinsic dignity of the person

The primary ethical foundation is the recognition of the disabled person's right to be treated with full respect and dignity as a human being, and the principle of autonomy refers to the respect due for fundamental human rights, including that of self-determination. This assertion is based on the consideration that every individual has an intrinsic dignity by the mere fact of being a human being (22). This ethical requirement of bioethical personalism is explicitly recalled by the UN Convention on the Rights of Persons with Disabilities, where the principle of respect for intrinsic dignity and individual autonomy is enshrined, including through the promotion of habilitative and rehabilitative interventions aimed at the full inclusion and participation of disabled people in all areas of life (23).

The principle of individual autonomy and respect for the intrinsic dignity of the person are also affirmed by the High Level Expert Group on AI, established by the European Commission in 2018. For an AI to be trustworthy, it is imperative to ensure that the humans interacting with its systems can retain their full and effective autonomy. These systems must be designed to enhance and integrate human cognitive, social and cultural capabilities (24). The explicit reference to AI as a tool for integrating human cognitive, social and cultural capabilities is consistent with the “biopsychosocial” approach that underpins the concept of disability on which the International Classification of Functioning, Disability and Health (ICF) was developed (25). The development of the concept of disability, compared to what was previously established in the International Classification of Impairments, Disabilities, and Handicaps (26), shows a transition from a functionalist definition, centered on the direct consequences of a disease, to the recognition of disability as the result of a complex interaction between an individual's state of health, personal factors and socio-environmental influences (27).

It is therefore necessary for AI systems to be integrated within habilitative and rehabilitative interventions in which the biological, individual and social dimensions of disability are fully grasped. These principles can certainly be unanimously recognized and agreed upon, but in the real world, AI systems with regard to disabled people can be helpful but also a source of frustration, they can fulfill the promises for which they were designed but also be a source of disappointment (28).

Specifically, we will address the bioethical issues related to the manner in which the use of an advanced robotic system could undermine the principle of autonomy of the disabled person with regard to the learning and data processing phases, which we will only subdivide to exemplify the exposition even though they are closely linked and interconnected.

With regard to the data processing phase, we need to make explicit what we mean by decision, how a patient confronted with therapeutic alternatives decides to choose one over another. The decision-making process, which is by no means mechanical, is the result of balancing the knowledge possessed and the possible predictions that can be inferred from it (29).

The question is to ask what knowledge possessed and what predictions can a patient refer to if specific characteristics of many AI technologies are opacity (“black box effect”), complexity, unpredictability and partially autonomous behavior (30).

The latest Machine Learning (ML) architectures are so intricate that they are able to anticipate and produce data without requiring prior understanding of causality and relationships between inputs and outputs, making it difficult for users to understand the process by which an artificial intelligence system translates data into decisions (31, 32).

As far as the data acquisition phase is concerned, it must be considered that ML comprises various computational methods through which AI systems can gradually build an accurate data model to support specific tasks, such as classification, clustering or regression. Classification identifies the correct category for each record, regression estimates the correct value of a continuous variable, while clustering groups similar instances into distinct groups, called clusters (33). However, an over- or under-representation of certain populations and sub-populations in the data in which the AI learns, or distortions at the level of the society to which the data refer, may be at the root of significant criticalities in the process of learning from data (34). Therefore, the processing of data through these techniques may not take into account the variations that a particular patient may present, resulting in a phenomenon known as “algorithmic discrimination” (35). There is a risk that by classifying or stratifying patients into groups or subgroups based on their personal profiles, discriminatory or stigmatizing decisions are made to the exclusion of health considerations and based only on certain profiles and criteria unrelated to health. The clustering of a disabled human being may not consider the implications that a robot-assisted habilitation or rehabilitation has on that specific person, not only in terms of physical health and psychological wellbeing, but also with regard to their daily activities, personal relationships and the effect on relationships in a specific social and environmental context. This approach would configure a functionalist and paradoxically anti-historical approach to disability, excluding the taking into account of the totality of the person. On the opposite, any habilitative or rehabilitative intervention is not just a set of therapeutic interventions aimed at the physical dimension of the body, even if advanced as in the case of robotics, but implies a human approach that takes into account the dignity and rights of the disabled person. This process is not only about restoring function, but also about establishing a human relationship with the vulnerable patient, actively involving him or her in the recovery process (36). Although these principles are crucial for any form of habilitation or rehabilitation, they assume even greater importance when using cybernetic physical systems (CPS), which have the potential to redefine our conception of a healthy body, being directly integrated or implanted in the human body (37). Rehabilitative intervention, while focusing on the damaged body, also has a broader impact on personality, considering the individual's familial, social, and work context. Through the body, individuals express themselves and interact with the world. Any somatic damage affects the psyche and personal identity, just as psychological alterations influence body perception and relationships.

However, the explainability and interpretability of AI systems should not be at odds with predictive and diagnostic accuracy. On the contrary, when intelligently integrated, they can significantly improve the trust, adoption and overall performance of AI systems. To this end, to ensure accountability and autonomy in medicine, regulations should limit the use of machine learning systems to specific empirically validated tasks, testing their robustness in real-world settings and comparing their performance with standard alternatives in prospective studies (38).

For the first time in the long history of medical sciences, the incorporation of generative AI in robotic systems allows for decisions that are completely independent of human input. Patients must be informed about this aspect and its potential consequences to respect and protect their right to self-determination. Established medico-legal literature shows that respecting and protecting this right is achieved when consent is informed, meaning it is preceded by detailed information appropriate to the patient's level of understanding, both in content and presentation of biological reality; and when consent is conscious, meaning it derives from clear and comprehensive information regarding the diagnostic-therapeutic approach, prognosis, presumed effects of the treatment, risks, and potential complications of the therapy. This principle is now well-established not only in scientific and legal literature but also in practice, to the extent that some medical and scientific publications design a “patient page” to provide medical information in simple and understandable language, aiming to educate and inform patients about specific health conditions, treatments, medical procedures, or health-related topics.

In order to guarantee the autonomy of the person and the expression of that autonomy in specific needs and individual preferences, it will be necessary for physicians to request specific consent from their patients. It is essential to ensure that the information provided to the patient is accurate, complete and understandable, as this contributes to a full understanding of the patient's health condition and available treatment options, thus enabling informed participation in medical decisions. The foundation for a sound decision-making process, which is based on knowledge and rational confidence in the expected results of a treatment, requires above all a comprehensive understanding of the AI system that must be explainable. To ensure the technical comprehensibility of an AI system, humans must be able to understand and monitor the decisions made by the system and receive information on the logic used in setting up an automated decision-making process as well as the possible consequences (39).

In this innovative area of healthcare, it is imperative for truly informed consent that physicians inform, and patients understand. We propose the following decalogue: (a) the functioning and reliability of AI systems. The information must be detailed and appropriate to the patient's level of understanding, both in content and in methods of presentation and representation of reality; (b) the limitations and potential errors of such systems; (c) how to manage potential conflicts between the human being and AI; (d) the security of the patient's personal information; (e) validation of the AI system's functioning; (f) the features of the patient population whose data were used to develop the algorithm and the possibility that an algorithm will perform less well in populations on which it has not been tested; (g) the limitations of human control and the supervisory possibilities on the robotic system; (h) the availability of sustainable access to maintenance, enhancements, software updates; (i) the ability to use third parties for essential services if the original provider is no longer available; (l) the risks associated with hacking, deactivation or memory erasure of CPS embedded in the human body (40, 41). A crucial interaction between medical doctors and clinical engineers could emerge to provide patients with precise and comprehensible information about AI. Physicians, with their clinical experience and understanding of patients' needs, collaborate with clinical engineers who possess technical expertise in AI. Together, they can translate the complex technical aspects of AI into easily understandable terms, ensuring that the information is detailed, accurate, and appropriate to the patient's level of understanding. This synergy would allow AI to be presented not only as an advanced diagnostic tool but also as an integrated element in the care pathway, enhancing patient trust and acceptance.

Every disabled human being undergoing habilitative or rehabilitative treatment with the use of robotics must be considered in his or her uniqueness. A high-tech context with complex terminologies and new healthcare procedures can compromise the patient's autonomy, transforming him or her from an active and dignified “subject” to an “object” of medical treatment. It is essential to adopt an approach to robotics and AI that respects the unique and inalienable moral status of the human being (42). Moreover, the use of robotics in disabilities may increase human vulnerability, creating psychological and social dependencies on robots as caregivers. The anthropomorphization of robots can generate emotional bonds and dependencies, particularly for fragile individuals such as people with disabilities (22, 43).

## Impact on relationships with healthcare workers

We recognize in technology a formal dynamic that represents a collective enterprise continually advancing according to its own laws of motion, and a substantive content consisting of the resources placed at the service of human beings, the potential and capacities it confers, the new goals proposed or imposed, and the changes in the modes of human action and behavior (44). For each of the aforementioned elements, it would be possible to detect and address ethical issues related to the use of technology in healthcare; however, in order to investigate the impact on the relationship between the disabled person and the Healthcare Workers (HWs), we will only dwell on the substantive content and ask the question: what must be done or not done for the human being to remain a human being?

The question concerning the impact of robotics on the relationship between patient and HWs arises mainly for robot-assisted home rehabilitation and therapeutic robots capable of simulating social interactions (15, 17). In these areas, the greatest risks could arise from over-reliance on new technologies, the emergence of new forms of addiction and the way patients self-manage their health condition.

The introduction of robotics and AI may distort the relationship between HWs and the impaired person, with a real risk of over-reliance on technology (41). This risk concerns patients, but also all HWs, including physicians. While it is important to build trustworthy AI systems, it is equally important to take safeguards to prevent overconfidence in the AI system or overreliance on it in work processes (24).

Indeed, the use of socially assistive robot (SAR) technology in elderly care has shown improvements in emotional and social wellbeing, especially in group settings, although their superiority over soft toys or placebo robots is debated. The effectiveness in cognitive training is acknowledged, but further studies are needed to confirm the benefits in dementia and to demonstrate clinical utility in physiological therapy (45). Another aspect to consider is the impact on relationships from an econometric perspective, considering that HWs are currently challenged to understand, prioritize, and deliver fundamental care, while healthcare organizations face shortages of qualified personnel and difficulties in mobilizing human resources. Technological integration can exacerbate or alleviate these challenges and calls for HWs to take strategic action to address exponential technological growth and ensure the provision of fundamental care (46).

Therefore, it is necessary to introduce the use of robots and robotic technology with foresight, developing specific guidelines to improve the lives of the elderly, reduce their dependency, and create greater opportunities for social interaction (47). At the same time, through a values-centered approach to design, engineers should incorporate reciprocity as a fundamental value in human-robot interaction, promoting critical dialogue to manage ethical risks and enhance the social effectiveness of robots (48, 49).

Considering the substantial diversity of opinions and ethical arguments, as well as the lack of consensus on the use of assistive robots, there is a need for on-going and contextual reflective evaluation (50).

The High-Level Expert Group on Artificial Intelligence envisages a horizon in which technology is not self-referential, but requires meditative thinking that can bring us face to face with the full domain of technology (51).

Therefore, in its Resolution of 17 February 2017, the European Parliament urged the Commission to submit a legislative proposal establishing civil law rules on robotics and AI, specifying that the introduction of robots in healthcare should not compromise the physician-patient relationship, but rather provide support in diagnosis and/or treatment to reduce the risk of human error and improve quality and life expectancy. In addition, the danger of dehumanization in care practices was recognized and the importance of preserving the role of caregivers for the inalienable human value in social interaction was emphasized (37). Subsequently, the Commission put forward the proposal of an “anthropocentric” IA model, which should include monitoring mechanisms, safety devices and traceability. Monitoring could be ensured through human involvement (human-in-the-loop), human supervision (human-on-the-loop), or human control (human-in-command). Safety devices should be integrated from the design phase in order to ensure the safety of AI systems in a traceable manner during each phase, with particular regard to the physical and mental protection of all persons involved (52).

Article 14 of the AI Act is focused on the human-machine interface and the primary role of the human being in decision-making. Before marketing, the provider must ensure the possibility of human surveillance and integrate it into the high-risk AI system. It is the human's job to monitor the operation of the system, to intervene promptly in the event of faults or malfunctions and to switch off the system if necessary. Furthermore, humans must be aware of the risk of “automation bias,” i.e., the tendency to over-rely on system output without critically evaluating it, and must therefore be prepared to interpret and, if necessary, ignore the output of the high-risk AI system (53).

A second issue to consider is that the use of robotics in the context of impairments could increase human vulnerability, giving rise to new forms of psychological dependency linked to the custom and support provided by robots as caregivers, and to social dependencies. In addition, the anthropomorphization of robots could arouse misleading feelings in humans, facilitating emotional bonds and dependencies, especially in fragile people (54, 55).

It has been argued that promoting the treatment of robots as social entities is ethically risky and misleading. Robots, being designed machines, cannot develop genuine social connections like living beings. Moreover, establishing social bonds with robots could generate a sense of moral obligation toward them, which could go against human wellbeing. In contrast, the relational approach to the sociality of robots challenges the idea that they are simply machines. It focuses on the dynamics and consequences of human-robot interactions rather than the categorical membership of robots. This perspective, which embraces the concept of social transaction, recognizes that inequalities and ethical harms arise from relationships, often influenced by stereotypes and essentialist attributions (56).

The implementation of “opt out” mechanisms to prevent the occurrence of technological dependency, similar to over-exposure warning systems, could be considered. Or one could limit the humanoid resemblance of robots to avoid an increase in emotional attachment beyond what is necessary for specific functions (20).

The widespread use of AI could lead to neo-paternalism due to permanent surveillance, undermining patient autonomy. Therefore, it is crucial to establish permissible levels of automation, always keeping a human in the decision-making loop to avoid fully automated decisions, and medical institutions should ensure that the time saved through new technologies is used to improve the doctor-patient relationship (57).

A third issue to consider is the potential impact of AI on the way patients manage their health. While some may welcome tools such as “chat bots” or health monitoring technologies, others may feel overwhelmed. Additional responsibility would be added to managing one's medication, improving nutrition, physical activity, wound care or self-administration (58).

The highlighted criticalities have a common response: what must be avoided is the substitution of the robot for the human relationship (20). In the context of the care relationship, the technical-scientific healthcare act takes on a profound moral value, as it gives the patient the security that the disease does not compromise his dignity and does not deprive his life experience of meaning (1). The biopsychosocial model provides an assessment of the state of health in which the complex relationships between body, mind, environment, social and cultural contexts are considered. Therefore, any habilitative or rehabilitative pathway becomes a dynamic project that presupposes the relationality and indispensability of human and professional qualities, such as empathic listening, the ability to interpret needs, the willingness to dialogue, the stimulation of therapeutic collaboration and the willingness to involve family members (59). Indeed, the reason that often prompts a person to seek medical assistance may not coincide with the main problem needing treatment, suggesting that a limited approach in the diagnostic, therapeutic and rehabilitation processes conducted by AI systems may reduce the opportunity to identify incidental results (60). Robotization can be a risk to human beings and their dignity to the extent that mechanized activities lead to a dehumanization of interpersonal relationships or cause a technological dependence of humans on machines. But it can also be, and in some cases already is, a great resource enabling significant advances in diagnosis, surgery, rehabilitation therapies and elderly care. Applications of robotics in medicine are radically transforming medical practice, offering more precise, efficient and personalized solutions (5). A positive, integrated approach is needed, as suggested by Topol in the three-component “deep medicine” model. Deep phenotyping' collects comprehensive data from various sources, including biological aspects such as DNA and microbiome. Deep learning' helps doctors in diagnosis, virtual medical coaching and patient safety, both in hospital and at home. Finally, “deep empathy” improves the connection between patients and doctors, with machines handling automatable tasks, allowing healthcare workers to focus on patient care (61).

Of course, this can only be achieved if healthcare institutions do not use the time savings made possible by a reduction in the administrative burden to move more patients through the system rather than allowing professionals to spend more time talking to and caring for their patients (62). And here again, meditative thinking is required in which the domain of technology is confronted with economic, political, institutional, and social dynamics and the ethos of medicine.

Recognizing the centrality of the human relationship and the complementarity of technologies requires that healthcare workers understand and effectively use AI and robotics to improve care. Training must include application knowledge, development of technical, communication and decision-making skills. However, the acquisition of these skills must not cause a “skill polarization” or discriminate against those who cannot learn them (63).

## Equity of access

The “digital divide” refers to the disparity between individuals, families, businesses and geographic areas in access to, use and skills in using digital technologies and this phenomenon can disproportionately affect marginalized communities and frail patients, potentially exacerbating existing health inequalities (18, 64).

It is interesting to note how the current debate on the ethical issues related to the use of robotics for disabled people partly echoes the discussions of the 1990's about the role of the Internet for disabled people, when it was questioned whether the spread of the Internet could improve opportunities for people with disabilities or increase inequalities (65). Starting from the premise of the indispensability of the Internet for carrying out daily activities, it was already highlighted in the past that, on one hand, disabled people faced difficulties such as disparities in access and the presence of specific barriers due to inaccessible design and incompatibility with assistive technologies. On the other hand, there was the necessity to leverage the Internet as a tool to promote the social inclusion of people with disabilities (66).

The Scientific Foresight Unit (STOA) of the European Parliament Research Service (EPRS) argued for equal opportunities and accessibility for all people in need of robot-provided healthcare and for the coordination of national legal systems so as to strengthen the principle of equality (67).

On 17 February 2017, the European Parliament in relation to restorative and enhancement interventions of the human body through AI, highlighted the importance of ensuring equal access of all citizens to such technological innovations, tools and interventions in accordance with the UN Convention on the Rights of Persons with Disabilities (37). In fact, the UN Convention, in Article 4, enshrines the need to take all appropriate measures to remove discrimination on the basis of disability by any person, organization or private enterprise, and promotes the research, development, availability and use of new technologies suitable for persons with disabilities, giving priority to technologies with the most accessible costs (23).

In the framework for trustworthy AI, equity is one of the four fundamental ethical principles and one of the seven key principles for the realization of trustworthy AI. The development, deployment and use of AI systems must be fair, just as the distribution of costs and benefits must be fair and equitable (24). The principle of equity as elaborated by the High-Level Expert Group on Artificial Intelligence was also recalled in the preamble of the recent AI Act aimed at ensuring the smooth functioning of the EU market through the harmonization of rules for the marketing, commissioning and use of AI systems (53).

Subsequently, the Commission proposed to develop an AI that serves people, with the main goal of improving human wellbeing. An “anthropocentric” AI should consider the full range of capabilities, skills and needs of human beings, ensuring that its applications are accessible to all. This implies adopting a universal design approach, aimed at ensuring equal access also for people with disabilities. Universal design must strive to eliminate barriers and create inclusive solutions that can be used by all, regardless of their different needs and abilities (52).

Ethical considerations on strategies to bridge the “digital divide” and ensure equal access to robotic tools for people with disabilities relate to resource management. Without addressing them, there is a risk that these tools will increase inequalities. Theories of justice agree on the need to allocate health resources but differ in their understanding of justice (68).

According to a functionalist approach, the allocation of economic and human resources would be closely linked to predictions of recovery in terms of efficiency and autonomy. Those who do not fit into these forecasts would not be entitled to habilitative or rehabilitative interventions using advanced technological tools. According to a contractualist approach, the ethical permissibility of a rehabilitative or habilitative intervention is closely related to the recovery of the full capacity for self-sufficiency, self-awareness and self-determination (20).

Both models appear to be unsuccessful because in arriving at an assessment of the ethical permissibility of resource allocation they disaggregate or do not consider the physical, human factor and social-environmental dimensions of disability. To successfully implement these technologies in the healthcare sector, it is crucial to deeply understand the local context, collaborate with stakeholders and adopt a customized approach to address specific challenges (69).

We believe, on the other hand, that the concept of equity according to the “biopsychosocial” model is based on the principle of equality. If disability is the product of the complex interaction between the body, the mind, the environment and the socio-cultural context, then every person has equal dignity and therefore the criteria assumed cannot be discriminatory. The core principle for guaranteeing equal access to robotics and AI in the context of disability is based on the idea of allocating resources and support in proportion to the severity of needs. This means that those with more complex needs or more severe disability conditions should receive more help and more advanced technological support. In practice, this involves several strategic actions. First, an accurate assessment of the individual needs of each person with a disability must be carried out. This assessment should consider various factors, such as the type and severity of the disability, functional limitations, age of the patient, the social and employment context, and the resources already available.

Then, based on this assessment, customized solutions can be designed and implemented. Another key aspect is to ensure the affordability of these technologies by developing financial support policies, such as subsidies, tax breaks, or insurance that cover the costs of such devices. This is particularly important for people with severe disabilities who often face greater economic and employment difficulties. Furthermore, it is crucial to promote research and innovation in the field of robotics and AI for disability, with the aim of developing increasingly effective and accessible solutions. This can include public and private funding for research projects, collaborations between universities, research institutions and technology companies, and the creation of training programmes for developers and engineers specialized in assistive technologies. Finally, societal awareness and education play a key role. It is important that the public, health professionals, educators and policy makers are aware of the potential of robotics and AI in improving the quality of life of people with disabilities. This can be achieved through information campaigns, training courses, and the sharing of good practices.

## Conclusions

People with disabilities are an expected user group of many products and processes using AI, including assistive devices and technologies. Currently, there is a lack of an ethical reference to guide interactions between disabled people and AI, as the focus is mainly on product usability. However, there may be many critical ethical issues related to the use of AI and robotics. We believe that a fundamental starting point is the need to integrate AI systems into habilitative and rehabilitative interventions, in line with the biopsychosocial model, to ensure that the biological, individual and social dimensions of disability are not neglected. Based on this assumption, the protection of the principle of autonomy and respect for the intrinsic dignity of the person is achieved by providing the patient with accurate, complete and easily comprehensible information. This approach fosters a clear understanding of one's own health condition and the actual significance of the use, limitations and potential of AI and robotics for rehabilitation purposes. To this purpose, it is imperative to recognize the centrality of the human relationship and the complementarity of these advanced technological tools. The use of robotics and AI should not be used to reduce the delivery of high-quality care provided by qualified rehabilitation professionals. It is also necessary to assess the impact they could have on the availability and utilization of existing rehabilitation services. Furthermore, the biopsychosocial concept of disability dictates that every person has the right to equal access to care. Therefore, in order to design and implement customized solutions and ensure affordability it will be necessary to assess individual needs, the severity of the disability the functional limitations, the social and work context, and the resources already available. Moreover, in addition to involving and considering all relevant stakeholders throughout the process, it is crucial to ensure equal treatment and access through inclusive design processes, without waiting until the testing or evaluation phase to involve disabled people.

Increased use of embodied artificial intelligence in healthcare requires careful exploration of the impacts on several levels. It is crucial to assess how AI influences decision-making processes. Research should examine whether AI provides effective support while also ensuring the safety and effectiveness of treatments. Human interactions in healthcare are equally crucial. The introduction of AI could change the nature of interactions between patients and HWs, affecting the quality of care and the level of mutual trust. It is relevant to study whether AI could improve communication and collaboration between the actors involved in patient care or whether, on the contrary, it could create emotional distance or disconnection. Finally, the risks of discrimination in healthcare require special attention. Research must examine whether AI may introduce or amplify biases that could lead to unfair or discriminatory treatment. Vigilance and implementation of corrective measures are essential to ensure that AI is used in an equitable manner and does not harm the quality and equity of healthcare.
